# Assessment of the Influence of Antisolvent 3D Printing Conditions on the Mechanical and Biological Properties of Poly(lactic-co-glycolic) Acid Scaffolds

**DOI:** 10.3390/polym17040501

**Published:** 2025-02-14

**Authors:** Anton V. Mironov, Ekaterina M. Trifanova, Tatyana B. Bukharova, Andrey V. Vasilyev, Viktoria O. Chernomyrdina, Irina A. Nedorubova, Valeriya S. Kuznetsova, Andrey G. Dunaev, Vladimir K. Popov, Anatoly A. Kulakov, Fedor F. Losev, Dmitry V. Goldshtein

**Affiliations:** 1 Central Research Institute of Dental and Maxillofacial Surgery, 119021 Moscow, Russia; em.trifanova@gmail.com (E.M.T.); vav-stom@yandex.ru (A.V.V.); victoria-mok@yandex.ru (V.O.C.); tilia7@yandex.ru (V.S.K.); dunaewan@gmail.com (A.G.D.);; 2 NRC «Kurchatov Institute», 119333 Moscow, Russia; vladikarpopov@gmail.com (V.K.P.);; 3 Research Centre for Medical Genetics, 115522 Moscow, Russia; bukharova-rmt@yandex.ru (T.B.B.); irina0140@gmail.com (I.A.N.); dvgoldshtein@gmail.com (D.V.G.)

**Keywords:** scaffold, poly(lactic-co-glycolic) acid, 3D printing, microstructure, multipotent mesenchymal stromal cells, mechanical properties, tissue engineering

## Abstract

This paper describes an evaluation of the mechanical and biological properties of highly porous, biocompatible poly(lactic-co-glycolic acid) (PLGA) scaffolds produced using the antisolvent 3D printing technique under various forming conditions. The dependence of the scaffolds’ microstructure, PLGA molecular weight distribution, and cell adhesion properties on temperature and injection nozzle diameter was evaluated. All samples consisted of fibers with different inner polymer distributions formed by specific radial, highly porous structures with a mean pore length of less than 50 μm and a diameter below 10 μm. The microstructure formed using a nozzle with a diameter of 160 μm showed a moderate correlation with printing temperature, while for the 330 μm nozzle, there was no significant difference in microstructures formed at different temperatures. Scaffolds produced at lower temperatures of 4 °C with a thin nozzle showed better compression load characteristics in terms of strength. In contrast, a larger nozzle allowed the production of a PLGA structure with improved elasticity. A 10–17% change in the molecular weight of PLGA was observed during printing, but no influence on biological properties was found. All types of PLGA scaffolds tested demonstrated good biocompatibility and promoted cell adhesion compared to the control.

## 1. Introduction

In traumatology, orthopedics, maxillofacial surgery, and dentistry, there are extensive bone defects requiring restoration [1,2,3]. The use of bioactive materials in combination with additive technologies is one of the most advanced approaches to such challenges [4]. The key component of an implantable bioactive framework is a bioresorbable material that serves as a temporary support for the natural extracellular matrix, helping to maintain tissue volume and shape. This material also promotes the migration, proliferation, and differentiation of cells, making it essential to choose a frame structure based on the mechanical and morphological properties of the native tissues being restored [5]. The requirements for such materials are fully met by biocompatible polymers capable of forming highly porous structures with an interconnected network of channels for blood vessel ingrowth, cell attachment, and proliferation. Polymers from the homologous series of aliphatic polyesters (polylactic acid, poly(lactic-co-glycolic) acid, poly(ε-caprolactone), and their copolymers) are among the best-studied synthetic materials of suitable physicochemical and mechanical properties for the fabrication of tissue-engineered constructs [6,7,8]. They are biocompatible, capable of controlled biodegradation, approved for use in clinical practice, and widely used in biomedical research [9,10]. Traditionally, solutions or melts of these polymers have been used for the fabrication of scaffolds [11,12,13]. Some of the well-known major drawbacks of conventional methods for fabricating polymer-based scaffolds often involve the use of high temperatures (higher than 40 °C) or hazardous solvents [11] leading to low porosity and limited compatibility with bioactive components such as cells or growth factors [14]. To address this, biocompatible solvents like tetraglycol (TG) are employed. TG can dissolve many polyesters that are practically insoluble in water. Compared to the chlorinated solvents traditionally used for polyesters, TG has no cytotoxic properties and good thermodynamic compatibility with water [15]. It can be almost completely removed from the polymer solution in any water-containing medium, such as culture serum or intercellular fluid. The polymer scaffold formed during such phase separation can acquire a developed microstructure, including porosity [16,17]. Earlier, we proposed and implemented a method of antisolvent three-dimensional printing of biocompatible PLGA scaffolds for tissue engineering with a radially oriented microstructure and a system of interconnected pores [18,19,20]. We have demonstrated the significant influence of the temperature regime during antisolvent printing on the intensity of mass transfer processes, as well as on the structure of the resulting polymer material [19]. This technique enables the formation of scaffolds with intricate microstructures and tunable porosity by precipitating PLGA from a biocompatible solvent into a water-based antisolvent system. However, it has been shown that antisolvent-printed PLGA scaffolds have some limitations, the most notable of which is poor mechanical properties [21].

In this study, we explore a combined approach to forming the micro- and macrostructure of PLGA scaffolds by varying structuring temperatures. For this, we combine the antisolvent printing method with a previously developed three-dimensional cryoprinting technique for hydrogels. This technique allows for control over material flowability and prevents fiber deformation under significant temperature gradients, improving spatial resolution [22,23]. This study aims to investigate the effects of varying printing conditions on the mechanical, microstructural, and biological properties of PLGA scaffolds produced through an antisolvent 3D printing method. By analyzing the influence of temperature and nozzle diameter, this work seeks to establish a framework for designing scaffolds with enhanced performance for bone tissue engineering.

## 2. Materials and Methods

### 2.1. Material

PLGA with a 75/25 lactide/glycolide ratio and a viscosity of 0.7 dL/g (Purasorb PDLG7507, Corbion PURAC Biochem, Gorinchem, The Netherlands) served as the base polymer. A 10 wt.% polymer solution was prepared by dissolving 1 g of PLGA in 9 g of tetraglycol (MQ200 purity, Sigma-Aldrich, St. Louis, MO, USA). The dissolution process occurred for 2 days at room temperature, employing a magnetic stirrer (DLab FlatSpin, Beijing, China) for thorough homogenization.

### 2.2. Three-Dimensional Printing

**Figure 1:**
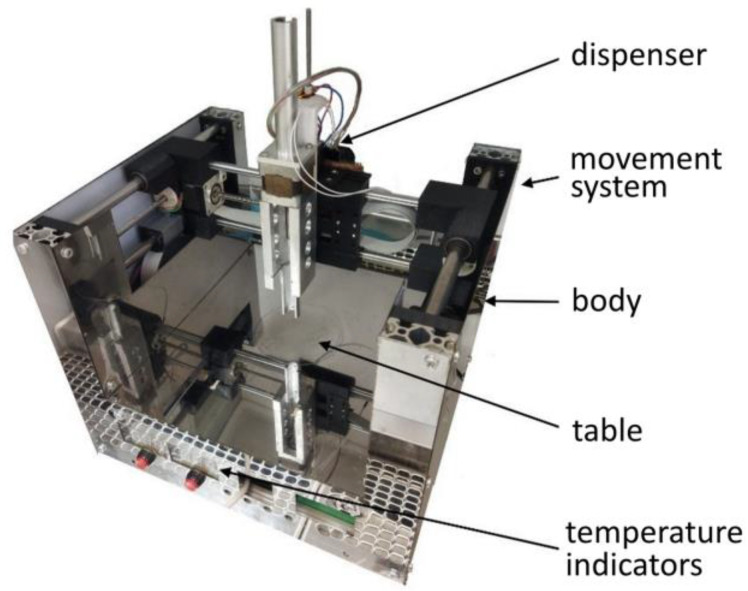
Laboratory 3D printer for antisolvent cryoprinting.

The production of the three-dimensional PLGA scaffolds was carried out on a custom-built 3D printer (Federal Scientific Center “Crystallography and Photonics” of the Russian Academy of Sciences) (Figure 1) controlled with RepetierHost software (v2.2.4, Willich, Germany). The basis of the printer is a motorized system that provides precise movement of the stepper motor driven syringe dispenser in three dimensions. During the printing process, the polymer solution was deposited on distilled water at coordinates specified in the three-dimensional (3D) digital model. The 3D structure was formed by the method of antisolvent precipitation through the diffusion of tetraglycol from the polymer solution into water.

The polymer solution was loaded into a 2 mL sterile syringe fitted with a stainless-steel nozzle. Two nozzle diameters, 160 µm and 330 µm, were employed to examine their effects on scaffold properties. To ensure sterility, the syringe was exposed to UV light at 240 nm wavelength for 10 min using a 40 W sterilization setup. Afterward, the syringe was positioned in the 3D printer’s dispenser. A sterile 120 mm plastic Petri dish filled with distilled water was placed on the printer table. The temperature was automatically maintained at 4 °C or 25 °C using 3D printer thermoelectric converters (TEC 12,708 type, CryoTherm, St. Petersburg, Russia). A digital model of a 3- and 5-layer lattice disc, each 5 mm in diameter, was used to prepare the G-code for the 3D printer using Cura slicer software ver.4.12 (Ultimaker, Utrecht, The Netherlands). The printing speed selection was limited by the maximum dispenser pressure and dependent on the nozzle diameter and temperature. To obtain a stable printing process, the flow rate was precisely controlled by the G-code and was as high as 0.02 μL/s for the 160 μm nozzle and 0.17 μL/s for the 330 μm nozzle. The layer height was set at 150 μm for the 160 μm nozzle and 330 μm for the 330 μm nozzle. The printing parameters and short sample characterization are provided in Table 1.

**Figure 2:**
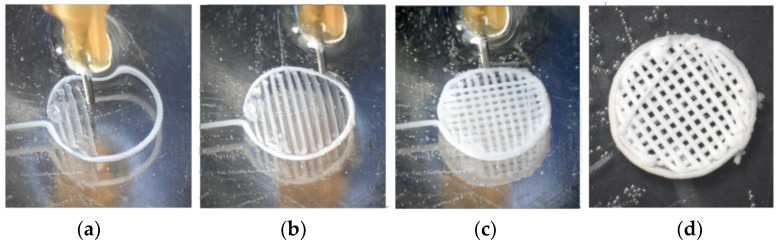
PLGA scaffold 3D printing (**a**–**c**) and solidifying (**d**).

The resulting polymer samples were kept in an overabundant amount of distilled water for 24 h. Afterward, the samples were rinsed with 95% ethyl alcohol and dried in air for another 24 h at room temperature (Figure 2).

### 2.3. Mechanical Testing

**Figure 3:**
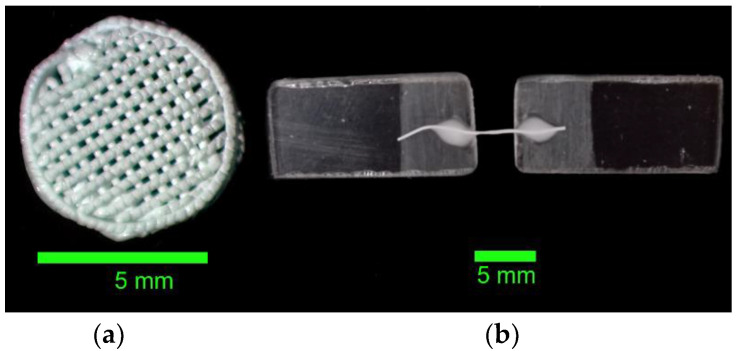
Mechanical test PLGA scaffold (**a**) and sample fiber (**b**).

The EZ-Test EZ-SX testing machine (Shimadzu, Kyoto, Japan) was used to carry out the mechanical property analysis. The two methods, compression and tensile tests, were used to investigate the mechanical properties of the specimens. Compression tests were performed on all 4 types of scaffold specimens described in Table 1. Then, 10 specimens were tested for each type of scaffold in both axial (5 pcs) and radial (5 pcs) tests to obtain the statistics. Each specimen was fixed between testing machine plates using fast-setting acrylic adhesive. Images of the specimens are shown in Figure 3a. To avoid the influence of infill direction on the radial compression test results, the samples were positioned to obtain a 45° angle between any scaffold infill fibers and the loading force direction. As it was difficult to correctly fix the scaffold in the mechanical tensile test stand, individual PLGA fibers were prepared to assess the tensile characterization. To prepare the tensile test sample, the segment of PLGA fiber with a diameter of 190 µm or 350 µm and a length of 15–20 mm was fixed between two acrylic plates as shown in Figure 3b. Then, a drop of water solution of poly(vinyl acetate) was used as a glue. After bond hardening, the sample was carefully tightened between the clamps of the testing machine.

To control the testing machine, TRAPEZIUM X software ver. 1.3.1 (Shimadzu, Kyoto, Japan) was used. The program managed the setting of all the mechanical test parameters (at a speed of 1 mm/min with no specific test limits). The Young’s moduli of the specimens were calculated from the linear regions of the stress–strain curves.

### 2.4. Molecular Weight Distribution of PLGA

The analysis of the molecular weight distribution of samples was conducted through gel permeation chromatography (GPC) on GPC/SEC “Stayer” (AO Akvilon, Moscow, Russia) with the refraction index detector and column Phenogel 5 µm 10^4^ Å (300 × 7.8 mm). The chromatograph was calibrated with polystyrene standards (Agilent Technologies Inc., Santa Clara, CA, USA). The molecular weight distribution in the initial PLGA granules was used as a reference in this analysis.

The polymer samples were dissolved in tetrahydrofuran (THF) (Sigma-Aldrich, St. Louis, MO, USA) (also used as the eluent in this system) at room temperature to obtain a solution with a sample concentration of 2.5 mg/mL. The injector loop (volume 200 µL) was filled with the resulting solution and, after stabilization of the baseline, molecular weight was measured at an eluent flow rate of 1 mL/min. The recorded data were automatically processed by the program “MultiChrom 1.6”. The molecular weight distribution analysis of each type of specimen was carried out on 8 specimens to obtain the statistics.

### 2.5. Microscopy

The macro-characteristics of the 3D scaffolds were analyzed using optical microscopy (Bresser microscope, Rhede, Germany) and scanning electron microscopy (SEM) (Phenom ProX microscope, Eindhoven, The Netherlands). A Scanning Electron Microscope (SEM) (Phenom ProX, Eindhoven, The Netherlands) was utilized to assess scaffold microstructure. Samples were analyzed under an accelerating voltage of 15 kV. Micrographs captured cross-sectional and surface morphologies, highlighting pore distribution and fiber architectonics. The samples were mounted on the microscope stage with conductive carbon tape.

### 2.6. Cell Culture

Rat adipose-derived stem cells (ADSCs) were cultured in a DMEM/F12 (PanEco, Moscow, Russia) medium supplemented with 10% fetal bovine serum (BioSera, Cholet, France), 0.584 mg/mL L-glutamine (PanEco, Moscow, Russia), and antibiotics (5000 units/mL penicillin and 5000 µg/mL streptomycin). The cells were maintained at 37 °C in a 5% CO_2_ atmosphere, with the growth medium being changed every 3 days.

### 2.7. Biocompatibility

To ensure sterility at every step, the scaffolds were preconditioned by two immersions in 70% ethanol for 10 min and physiological solution washes. To evaluate the cytocompatibility of the PLGA-based 3D scaffolds, rat ADSCs were seeded onto the bottom of 24-well plates with a Transwell system (8 μm pores, “SPL Lifesciences”, Naechon-Myeon, Republic of Korea). After 24 h, the scaffolds were transferred to the upper inserts and incubated for 14 days in the growth medium. At the end of the experiment, cell viability was evaluated using the MTT assay.

For cell adhesion analysis, PKH-26 staining (red fluorescent cell linker kit, Sigma-Aldrich, St. Louis, MO, USA) was used. PLGA-based 3D scaffolds were placed in the bottom of 24-well plates, and a cell suspension pre-stained with the red fluorescent dye PKH-26 (following the manufacturer’s instructions) was added on top. Cells were incubated with the scaffolds for 1 and 14 days, and at the end of the experiment, live cells were identified using the vital dye Calcein AM (0.5 µM, Biotium, Fremont, CA, USA) for 35 min at 37 °C. Apoptotic cells were detected with the fluorescent dye DAPI (4,6-diamidino-2-phenylindole) at 1 µg/mL for 10 min. The Lionheart FX automated microscope (Agilent BioTek, Santa Clara, CA, USA) was used to perform fluorescence microscopy analysis.

### 2.8. Statistics

The data were analyzed using GraphPad Prism software ver.10.4.0 (GraphPad Software, Inc., San Diego, CA, USA). The Shapiro–Wilk test assessed normality, and group comparisons were made using Student’s *t*-tests or Mann–Whitney U-tests. Statistical significance was set when the probability was less than 5% (*p* < 0.05). Values significantly different from the control ones were marked on the diagram with an asterisk (*) according to the American Physiological Association (APA) guidelines.

## 3. Results and Discussion

### 3.1. Formation of Three-Dimensional PLGA Scaffolds

**Figure 4:**
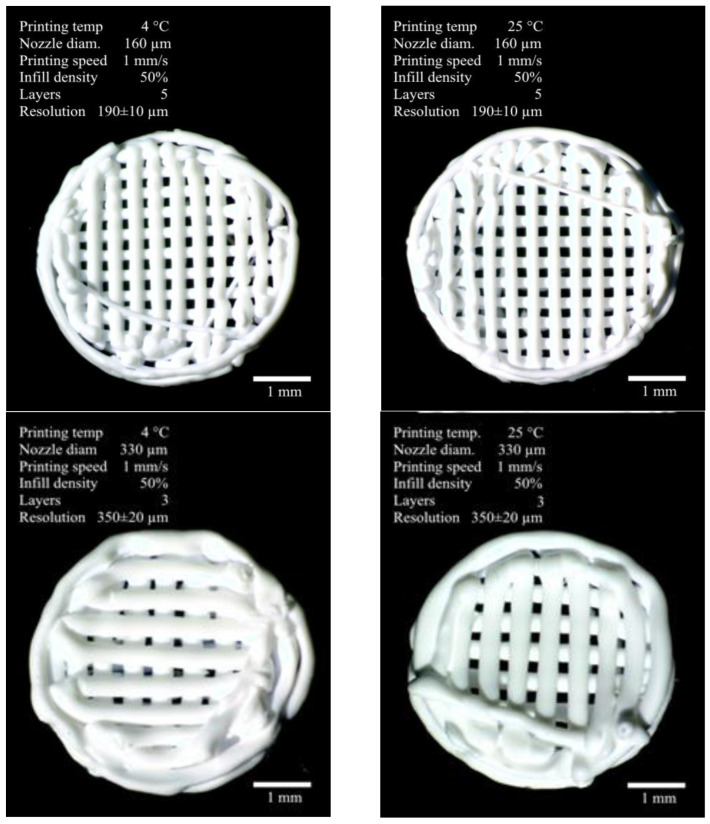
The appearance of scaffolds formed by antisolvent 3D printing. Optical microscopy.

Four series of experimental PLGA scaffold samples were formed using the method of antisolvent three-dimensional cryoprinting. Their appearance, fabrication parameters, and properties are shown in Figure 4. All the samples were white discs with a lattice structure of perpendicular chords stacked on top of each other, with the degree of filling varying depending on the thickness of the nozzle used.

It was shown that doubling the print nozzle diameter consistently led to a nearly twofold decrease in printing resolution. Additionally, there was a noticeable effect of jet expansion, likely of a relaxation nature, amounting to 19% for the 160 μm nozzle and 6% for the 330 μm nozzle. This indicates a greater macromolecule orientation force in the 160 μm channel compared to the 330 μm channel, even despite the six-times difference in flow rate (0.020 μL/s and 0.17 μL/s, respectively). On a macroscopic scale, the samples obtained under different conditions differed only in the filament thickness forming the structure. The average fiber diameter is 190 μm from the 160 μm nozzle and 350 μm from the 330 μm nozzle. The samples fully matched the digital model, with clearly distinguishable structural elements—the outer envelope and the grid filling.

### 3.2. Study of the PLGA Scaffold Microstructure

When developing bone tissue substitutes, it is important to take into account the porosity of the final product. It is usually recommended to use two types of porosity—micro and macro (>100 microns) [24]. Some papers present the results of studies of the properties of lattice frames with approximately the same diameters of fibers and pores [25]. It has been shown that PLA scaffolds have a rigid structure and can be used to replace the trabecular bone. PLGA is also successfully used for bone engineering in its pure form [4], as well as with the inclusion of hydroxyapatite [26].

As will be shown below, the antisolvent-printed PLGA scaffolds have pores in both ranges. Accordingly, there is reason to assume that they provide better cell attachment (microporosity) and the possibility of osteogenesis in general (macroporosity).

**Figure 5:**
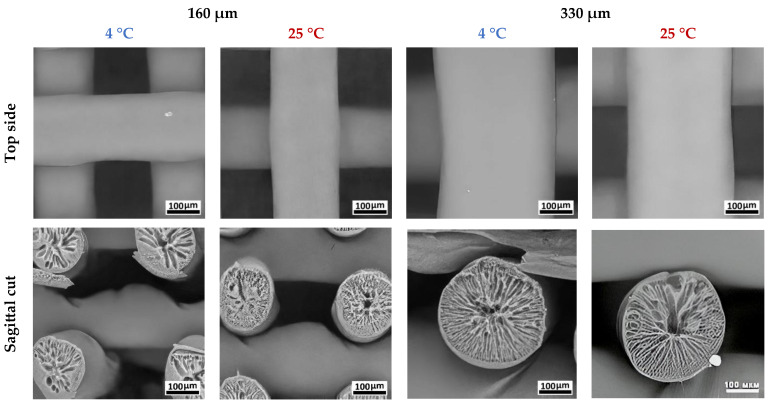
SEM images of structure and surface of antisolvent-printed PLGA scaffolds.

Previously, we showed that the architectonics of antisolvent-printed samples could depend on manufacturing conditions [19]. In the present study, it was found that the internal structure of the fibers forming the scaffold was not significantly different after alcohol treatment and had a porous structure with a radial orientation (Figure 5). As shown in the image, the outer surface of each fiber consists of a thin layer no thicker than 1 µm. The appearance of this internal structure is due to the formation of a countercurrent of solvent and antisolvent during the fiber solidification process. An anisotropic density distribution was observed in the structure of all fiber samples, with a tendency to form a hollow channel in the center of the fiber.

A comparative analysis of the microstructure of the samples showed differences in pore distribution across the fiber cross-section depending on printing conditions. As the temperature increased from 4 °C to 25 °C, a structure formed by large, radial, finger-like pores with characteristic dimensions of 30–50 μm was replaced by a network of smaller pores up to 30 μm in length (Figure 5). For fibers with a diameter of 190 μm, the difference in pore size formed at 4 °C and 25 °C was 30–50%. For fibers with a diameter of 350 µm, the differences in large pore size did not exceed 10%. The mean volume of a single pore formed during antisolvent printing directly depended on polymer precipitation time, as larger voids were a product of initial microphase merging. Higher temperatures led to faster polymer precipitation and blocked pore growth. At the same time, a larger depot of solvent in the case of thicker fibers allows the polymer solution to anticipate temperature rises and remain in the liquid state for longer, forming larger pores [15,19].

### 3.3. Study of the Mechanical Properties of PLGA Scaffolds

**Figure 6:**
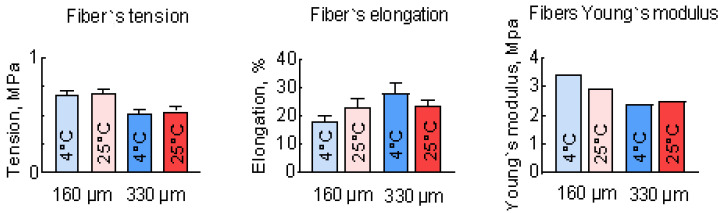
Mechanical properties of PLGA fibers formed by the antisolvent deposition method.

To investigate the tensile strength of the individual fibers, a tensile test was conducted. The results of these tests are shown in Figure 6. The Young’s modulus of these samples was calculated as the linear coefficient in the linear relationship between shear stress and strain. Thinner fibers showed greater rigidity, with a breaking force that was about 20% higher than that of thicker fibers. Temperature increases lead to a decrease in Young`s modulus and elongation at break, but this effect is not so pronounced for fibers with a diameter of 350 μm. These results are in good relation with the observed difference in the fibers’ inner structure (Figure 3), where the 350 μm fibers are much more uniform than the 190 μm fibers. The obtained results show that the tensile strength of the scaffold cannot exceed 0.7 MPa even if it has a monolithic macrostructure, which makes it suitable only for bone defect implantation with externally compensated or low tensile load.

**Figure 7:**
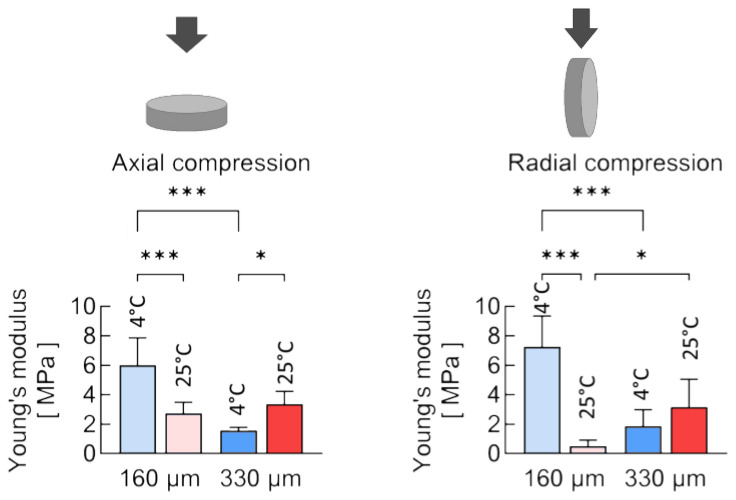
Young’s modulus at the compression of PLGA samples obtained under various conditions. * *p* < 0.05, *** *p* < 0.001.

Nevertheless, the mechanical properties of scaffolds depend not only on structural elements like fibers but also on their macrostructure. There are many different methods for assessing the strength and elasticity of materials, but for printed three-dimensional scaffolds mechanical compression tests are the most commonly applied [4,20,21]. Both axial and radial compression tests were conducted. These tests allowed a more comprehensive understanding of the mechanical properties of the scaffold as a whole. The results of the compression tests are presented in Figure 7. The scaffolds with 160 μm nozzle diameter produced at 4 °C had the highest Young’s modulus among the samples in the experiment for both axial and radial compression—6 ± 1.8 MPa and 7.2 ± 2 MPa, respectively. In contrast, for the samples with 330 μm at 4 °C, these values were 1.57 ± 0.17 MPa and 1.9 ± 0.7 MPa, respectively.

The changes in temperature conditions had little effect on the strength properties of PLGA samples formed by the antisolvent method. However, statistically significant differences in the relative elongation of samples formed at 4 °C and 25 °C were found, which could be explained by differences in scaffold porosity.

The presence of larger, elongated pores and an uneven distribution of voids in the samples obtained at 4 °C resulted in significantly greater material elongation when the internal structural elements were aligned with the applied force. In contrast, for the more uniform structure obtained at 25 °C, the pores compressed at a significantly lower strain in the sample.

The results of the study on individual fibers and scaffolds showed similar qualitative trends towards a decrease in strength and Young’s modulus as fiber thickness increased. However, there was no clear quantitative correlation between the microstructural characteristics of the scaffolds and their mechanical properties. This suggests that the macrostructure of the scaffold, including its shape and filling density, also significantly contributes to these properties. It is also important to note that comparisons between properties with different types of loads should be approached with caution. The mechanical properties of the investigated geometries of PLA scaffolds partially align with their intended applications. Even stiffer PLGA scaffolds seem to be not fully suitable for bone defects and need to be mechanically supported in some cases, as their strength is an order of magnitude lower than that of bone. At the same time, elastic scaffolds could be useful for soft tissue engineering. Another factor that could influence mechanical properties is the difference in the molecular weights of PLGA that occurred in the interaction with the hydrophilic solvent TG.

### 3.4. Molecular Mass Distribution of PLGA Scaffolds

**Figure 8:**
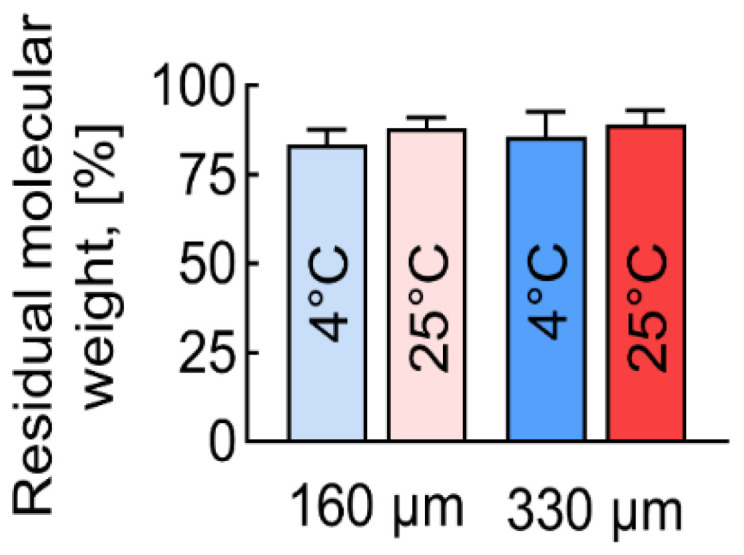
Loss of molecular weight (Mw) of PLGA after antisolvent 3D-printing process.

After antisolvent printing, the PLGA samples lost some of their molecular weight compared to the original PLGA (Figure 8). In all cases, a statistically significant decrease in the average molecular weight (Mw) and average number of molecular weight (Mn) of the molecules was detected. This decrease was due to the hydrolysis process of PLGA macromolecules.

The diameter of the fibers influenced the resulting molecular weight of the antisolvent 3D-printed PLGA. Fibers with a diameter of 190 microns (μm) showed a molecular mass loss that was 2–3% higher than those with a diameter of 350 μm. It can be assumed that the hydrolysis of the PLGA, promoted by the alcohol treatment, mainly occurs on the surface of the fibers, while the polymer in the fibers’ center remains intact. If this assumption was correct, then fibers with a larger volume-to-surface ratio should show a lower molecular mass decrease, which was observed.

Samples produced at lower temperatures showed a greater loss of molecular weight. The lower the printing temperature, the more molecular weight was lost, ranging from 10 to 15% at 25 °C to 15 to 18% at 4 °C.

Even after the antisolvent 3D printing process, the polydispersity (Pd) index of all the samples, including the initial PLGA granules used as a control, was below 2, which is typical for synthetic polymers (Table 2). However, a slight increase in Pd may indicate the presence of low-molecular-mass PLGA residues, which could affect the mechanical properties of the scaffold.

No direct relationship was observed between molecular weight and mechanical properties. Even an 18% decrease in PLGA’s molecular weight seemed to be too small to significantly affect properties and was likely masked by other factors such as temperature. Given the well-known fact of a loss of polymer strength with a decrease in its molecular weight, it can be concluded that the proposed method allows sufficient molecular weight to be maintained to keep appropriate mechanical and biological characteristics of the created scaffolds.

### 3.5. Biocompatibility

**Figure 9:**
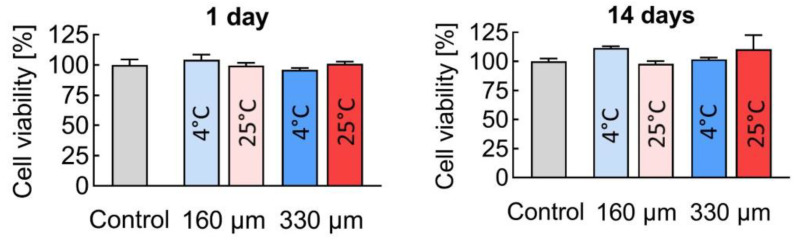
MTT test evaluation of the cytocompatibility of PLGA based 3D scaffolds after incubation with ADSCs.

Using the MTT assay, it was shown that during the first 24 h, no statistically significant differences in relative cell viability were observed. After 14 days, however, the number of viable cells in the presence of the scaffolds prepared with a 160 μm nozzle at 4 °C (Figure 9) even exceeded the control values, reaching 111.6 ± 1.5%.

**Figure 10:**
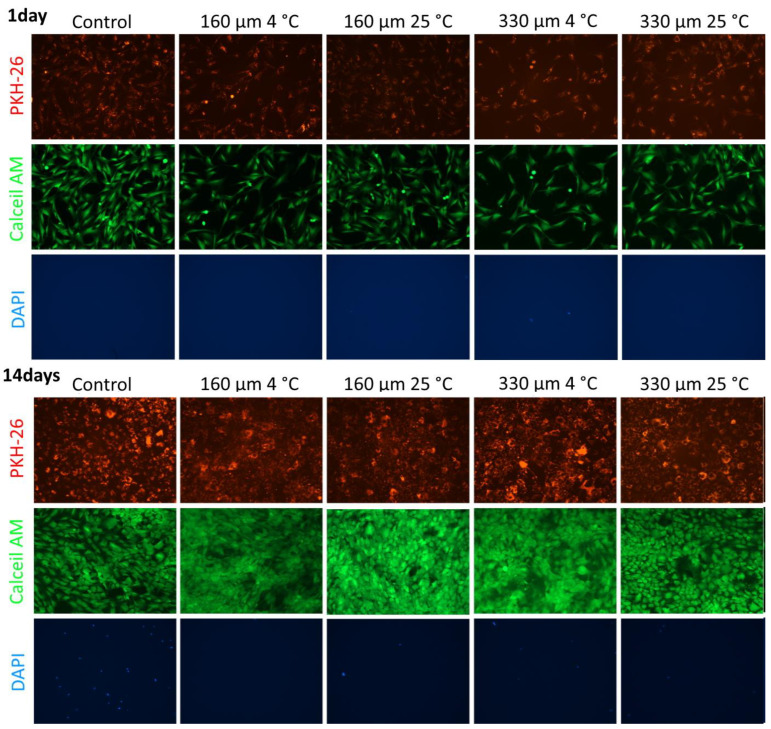
Evaluation of cytotoxicity of PLGA-based 3D scaffolds after incubation with ADSCs. Fluorescence microscopy; 10× magnification.

Live/dead cell staining revealed that no dead cells, as indicated by DAPI staining, were present after 24 h. After 14 days, there was a significant increase in cell density, with most cells remaining alive and stained with Calcein AM. A small number of dead cells were observed, but their presence was similar to that in the control group (Figure 10). These results demonstrate that the scaffolds exhibited high cytocompatibility.

**Figure 11:**
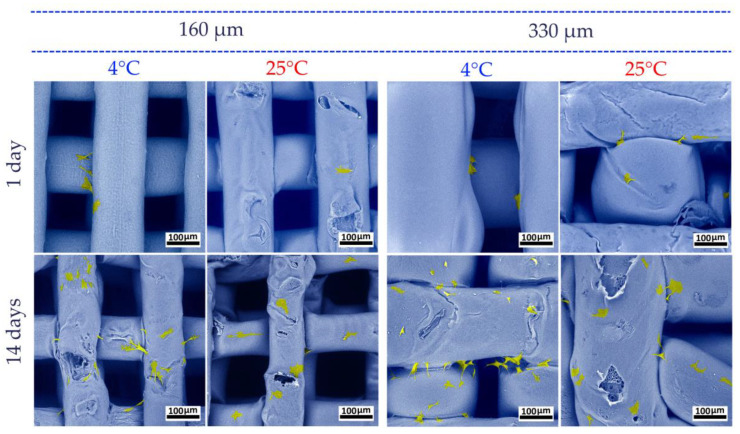
Cells attached to the scaffolds. SEM images after 1 day and after 14 days of culture. Blue—PLGA scaffold surface, yellow—cells.

A detailed analysis of cell morphology on the obtained scaffolds was carried out using SEM. It was shown that after 24 h, cells were tightly attached and spread on the surface of the materials (Figure 11). The ADSCs showed a characteristic morphology, with spreading cells exhibiting polygonal, spindle-, and fibroblast-like shapes. On day 14, a significant increase in the number of cells on the scaffolds was observed. The number of cells attached to the surface of samples printed at 4 °C was slightly higher than that of samples printed at 25 °C. It is suggested that the nature of the scaffold surface, which was formed with a more rigid structure at 4 °C, was more conducive to cell attachment. At the same time, no correlation of the cell viability with a difference in the molecular mass of PLGA scaffold samples was found.

Three-dimensional printing technology holds great promise for the production of scaffolds to replace extensive bone defects. It allows control over porosity, pore size, and geometry, creating conditions that mimic the microenvironment for cells and promote bone healing. Our study showed that the 3D printing mode—the temperature of printing and nozzle diameter—affected the microstructure and elastic modulus of the PLGA material, but did not significantly affect its strength and cytocompatibility.

The produced scaffolds were comparable in morphology and structure to human bone. The use of PLGA as a base allowed the creation of scaffolds that were capable of resorption and replacement by newly formed bone tissue. The obtained scaffolds had a smooth surface and an internal structure with a system of interconnected pores. There was a 10–15% decrease in PLGA molecular weight found after antisolvent 3D printing and treatment, but there was no evidence of systematic influence on scaffold properties. The Young’s modulus of the samples produced by the antisolvent method was 18–22 MPa, which was close to the Young’s modulus of bone tissue (15 MPa–32 GPa) [24,25]. The tensile strength of porous PLGA samples printed by the antisolvent method (0.3–0.42 MPa) was 1–2 orders of magnitude lower than the strength of bone tissue [25,26], but still enough for surgical manipulation and withstanding live tissue load. In vitro studies showed that the PLGA scaffolds obtained by the antisolvent 3D printing method had good biocompatibility. The macro- and microstructure of the scaffolds favor cell adhesion and proliferation. A comparison of the biocompatibility of the 3D-printed PLGA scaffolds did not show any significant effect of the forming conditions on cell survival.

## 4. Conclusions

The PLGA scaffolds for bone regeneration were successfully printed using an antisolvent 3D printing technique under different forming conditions. The internal structure of the scaffolds was analyzed, and it was found that the properties of PLGA scaffolds make them promising as a material for scaffolds. At this time, the method presented does not allow for the creation of a tissue regeneration scaffold with mechanical properties that perfectly mimic bone tissue and can withstand a full bone load in vivo. Nevertheless, the obtained PLA scaffold’s properties make it promising as a material for an artificial matrix for regenerating extensive defects in mechanically unloaded tissue.

The results highlight the role of printing parameters, such as temperature and nozzle diameter, in determining the structural, mechanical, and biological properties of the scaffolds. Scaffolds produced at a lower temperature of 4 °C and with a smaller nozzle diameter of 160 microns demonstrated better strength characteristics, while using a larger nozzle of 330 microns allowed the production of a porous PLGA structure with a lower Young’s modulus of 20%, which is potentially suitable for soft tissue defects. Despite a measurable reduction in molecular weight (10–18%) during the printing process, the scaffolds retained their mechanical integrity and biocompatibility. This indicates the reliability of the fabrication technique and its suitability for further application. Biocompatibility assessments confirmed high cell viability, cell adhesion, and proliferation rates, further validating the scaffolds’ potential for tissue engineering purposes.

Overall, this work provides a comprehensive framework for tuning scaffold properties through controlled printing parameters, paving the way for customized solutions in regenerative medicine.

## Figures and Tables

**Table 1:** Sample characterization and 3D printing parameters.

	Sample No.1	Sample No.2	Sample No.3	Sample No.4	
Material	poly(lactic-co-glycolic acid) Purasorb PDLG 7507				
Sample geometry *	lattice disc d = 5 mm, h = 1 mm				
Method	antisolvent 3D printing				
Nozzle diameter, µm	160		330		
Printing speed, mm/s **	1		2		
Layers number	5		3		
Flow rate, µL/s	0.020		0.171		
Printing resolution, µm ***	190 ± 10		350 ± 20		
Printing temperature, °C	4	25	4	25	

* d—sample diameter; h—sample height. **—maximum allowed speed. *** ± std.dev.

**Table 2:** Molecular weight (Mn) and polydispersity of antisolvent 3D-printed PLGA.

	Control	190 μm Fiber		350 μm Fiber	
		4 °C	25 °C	4 °C	25 °C
Mn	51,721	43,143	45,541	44,275	46,057
Mw	80,292	68,879	73,271	70,127	72,652
Pd	1.54	1.6	1.59	1.58	1.58

## Data Availability

The original contributions presented in the study are included in the article; further inquiries can be directed to the corresponding author.
